# Enterovirus 71 Antagonizes Antiviral Effects of Type III Interferon and Evades the Clearance of Intestinal Intraepithelial Lymphocytes

**DOI:** 10.3389/fmicb.2021.806084

**Published:** 2022-02-01

**Authors:** Yuanmin Dong, Jing Liu, Nan Lu, Cai Zhang

**Affiliations:** ^1^Institute of Immunopharmaceutical Sciences, School of Pharmaceutical Sciences, Cheeloo College of Medicine, Shandong University, Jinan, China; ^2^Key Laboratory of Special Pathogens and Biosafety, Wuhan Institute of Virology, Center for Biosafety Mega-Science, Chinese Academy of Sciences, Wuhan, China; ^3^Institute of Diagnostics, School of Medicine, Cheeloo College of Medicine, Shandong University, Jinan, China

**Keywords:** EV71, type III interferon, immune evasion, intestinal mucosal immunity, intestinal epithelial cells, intestinal intraepithelial lymphocytes, NK cells, NKG2D ligands

## Abstract

Enterovirus 71 (EV71) is the major pathogen causing severe neurological complications and hand, foot, and mouth disease. The intestinal mucosal immune system has a complete immune response and immune regulation mechanism, consisting of densely arranged monolayer intestinal epithelial cells (IECs) and intestinal intraepithelial lymphocytes (iIELs) distributed among the IECs, which constitute the first line of intestinal mucosa against infection of foreign pathogens. As an enterovirus, EV71 is transmitted by the intestinal tract; however, the mechanisms it uses to evade the immunosurveillance of the intestinal mucosal immune system are still incompletely clarified. The present study investigated how EV71 evades from recognizing and eliminating IECs, iIELs, and iNK cells. We found that EV71 infection induced a higher level of type III interferons (IFN-λ) than type I interferons (IFN-β) in IECs, and the addition of IFN-λ markedly restricted EV71 replication in IECs. These results indicate that IFN-λ plays a more important role in anti-EV71 intestinal infection. However, EV71 infection could markedly attenuate the antiviral responses of IFN-λ. Mechanistically, 2A protease (2Apro) and 3C protease (3Cpro) of EV71 inhibited the IFN-λ production and IFN-λ receptor expression and further decreased the response of IECs to IFN-λ. In addition, we found that EV71-infected IECs were less susceptible to the lysis of intestinal NK (iNK) cells and CD3^+^iIELs. We revealed that the viral 2Apro and 3Cpro could significantly reduce the expression of the ligands of natural killer group 2D (NKG2D) and promote the expression of PD-L1 on IECs, rendering them to evade the recognition and killing of iNK and CD3^+^iIELs. These results provide novel evasion mechanisms of EV71 from intestinal mucosal innate immunity and may give new insights into antiviral therapy.

## Introduction

Enterovirus 71 (EV71) belongs to the *Enterovirus* genus of the family *Picornaviridae*, which is transmitted through respiratory and gastrointestinal tracts, threatening the health of infants and children and causing severe infectious diseases in the Asia-Pacific region. EV71 is one of the main pathogens causing sporadic outbreaks of hand, foot, and mouth disease (HFMD). It mainly causes fever in children under 5 years old, accompanied by ulcers or rashes in the mouth, hands, and feet. In particular, it is generally self-limited but occasionally associated with severe symptoms of central nervous system (CNS) complications during acute infection, such as aseptic meningitis, encephalitis, myocarditis, and pulmonary edema (Solomon et al., 2010).

The viral particle of EV71, about 20∼30 nm diameter, consists of a non-enveloped icosahedral capsid surrounding a core of specific positive-strand RNA of approximately 7.5 kb in length, which encodes the structural protein (VP1, VP2, VP3, and VP4) and the non-structural proteins (2A, 2B, 2C, 3A, 3B, 3C, and 3D) (Jin et al., 2018). The structural protein VP1 mainly promotes infection by binding to specific receptors on the surface of host cells, including human scavenger receptor class B, member 2 (h-SCARB2), p-selection glycoprotein ligand-1 (PSGL-1), annexin II, and SA-linked glycan. Because EV71 is more likely to infect humans, the exogenous expression of human-SCARB2 or PSGL-1 could reverse non-permissive cell lines such as mouse fibroblast L929 to support EV71 infection instead and generate cytopathic effects. Also, EV71 clinical isolates could successfully infect the transgenic mouse model of expressing h-SCARB2 (Yang et al., 2019).

As the first line of host immune response defense, the innate immune system provides early and immediate defense against invasive pathogens (Thompson et al., 2011; Zhang et al., 2019; Shao et al., 2021). The diverse but conserved pathogen-associated molecular patterns (PAMPs) can be directly monitored and recognized by pattern recognition receptors (PRRs) to activate the host innate immune response, induce higher levels of interferons (IFNs) and cytokines, and effectively limit infection (Onomoto et al., 2021). Subsequently, they trigger appropriate signals to activate adaptive immune responses for the clearance of pathogens. IFNs play a vital role in the mammalian responses to viral infection, controlling viral replication and the spread of viruses (Raftery and Stevenson, 2017; Zhao and Fessler, 2020). IFNs mainly include type I IFN, type II IFN, and type III IFN (IFN-III). Type I IFN is widely expressed in mammalian cells and plays a key role against various types of virus infection. Type II IFN (IFN-γ) is produced by activated T cells or NK cells and has immunomodulatory and antiviral activities. IFN-III, consisting of IFN-λ1, IFN-λ2, IFN-λ3, and IFN-λ4, performs a crucial antiviral immune response and in the early stages of viral infection (Wells and Coyne, 2018; Lazear et al., 2019). The PRRs such as TLRs, RIG-I, and MDA5 can quickly recognize the dsRNA component of EV71, initiate downstream signaling pathways, such as the phosphorylation of IRF-3, IRF-7, and NF-κB, and further induce the production of IFNs. IFNs bind to specific receptors on the cell membrane through autocrine or paracrine, inducing many ISGs and other antiviral proteins. IFNs also have immunomodulatory functions, such as promoting the expression of MHC I on various types of cells, promoting the expression of costimulatory molecules on the surface of DCs, promoting the proliferation of T cells, and stimulating the antiviral function of adaptive immunity (Ank et al., 2006; Wedekind et al., 2020).

The host’s innate immune response is the most effective way to fight against viral infections. However, EV71 has evolved a series of strategies to interfere with the antiviral function of innate immunity, thus evading host innate immune surveillance. EV71 proteases 2A (2Apro) and 3C (3Cpro) are cysteine proteases belonging to the chymotrypsin-related endopeptidase proteases family that promote infection by interfering with the adaptor molecules that are involved in IFNs production and response (Rasti et al., 2019). In addition, 3Cpro of EV71 inhibits type I IFNs induction by targeting cytosolic helicase RIG-I by destroying RIG-I’s binding to mitochondrial antiviral signal protein (MAVS), inhibiting the activation and nuclear translocation of IRF3, and finally resulting in the inhibition of host antiviral response (Lei et al., 2010). EV71 2Apro can cleave MDA5 and MAVS to prevent PRRs from recognizing invading viruses (Wang et al., 2013; Feng et al., 2014). EV71 3Cpro can also cleave the adaptor protein TRIF and inhibit the activation of IRF3 and NF-κB, ultimately impairing IFNs production in response to TLR3 activation (Lei et al., 2011).

EV71 is an enterovirus, and the human gastrointestinal is the primary invasion portal. The intestinal mucosal immune barrier consists of densely arranged monolayer intestinal epithelial cells (IECs) that provide a barrier against harmful pathogens and the vast majority of intraepithelial lymphocytes (iIELs) located between IECs, forming the first line of the intestinal mucosa (Ma et al., 2019; Brown and Esterhazy, 2021; Losurdo et al., 2021). IECs express a variety of PRRs, such as TLRs and RLRs, which can directly monitor and recognize PAMPs (Peterson and Artis, 2014; Yang and Yan, 2021). The iIELs patrol between IECs, express various natural killer receptors (NKRs), and have a cytolytic property by producing granzyme and perforin. They can identify infected IECs in time and exert NK-like killing activity properties (Hayday et al., 2001). Intestinal NK (iNK) cells are important members of the intestinal intraepithelial lymphocytes, which express NKp44 in humans, or NKp46 and NK1.1 in mice (Olivares-Villagomez and Van Kaer, 2018). They can identify the pathogenic bacteria and viruses in time and produce proinflammatory factors such as IFN-γ and TNF-α (Peterson and Artis, 2014). The iNK cells play an essential role in the fight against intestinal bacterial and viral infection. They can inhibit HIV infection in intestinal mucosa for the long term (Sips et al., 2012). In addition, iNK cells have been shown to play a vital role in controlling murine intestinal coronavirus infection (Carman et al., 1986). Therefore, IECs, iIELs, and iNK cells constitute the first defense line to maintain the intestinal immune homeostasis and resist foreign pathogens. However, the mechanisms of EV71 escaping from the antiviral of the intestinal mucosal immune system are still incompletely clarified.

This study provides evidence of how EV71 evades from recognizing and eliminating IECs, iNK cells, and CD3^+^iIELs. We found that IFN-λ played a more important role in anti-EV71 intestinal infection compared with IFN-β. However, EV71 could markedly attenuate the antiviral responses of IFN-λ by its 2Apro and 3Cpro, which inhibited the IFN-λ production and IFN-λ receptor expression, and further decreased the response of IECs to IFN-λ. Further studies revealed that the viral 2Apro and 3Cpro could significantly reduce the expression of ligands of natural killer group 2D (NKG2D) and up-regulate the expression of PD-L1 on IECs, rendering them to evade the recognition and killing of iNK cells and CD3^+^iIELs.

## Materials and Methods

### Virus, Cells, and Reagents

Enterovirus 71 BrCr strain was provided by Wuhan Institute of Virology, Chinese Academy of Sciences. African green monkey kidney cells (Vero) were grown in Dulbecco’s modified Eagle’s minimum essential medium (DMEM) (Gibco). EV71 was propagated in Vero cells. Human colorectal adenocarcinoma HT29 cells were grown in RPMI-1640 medium (Gibco) supplemented with 10% FBS. Jurkat T cells were obtained from ATCC and cultured in RPMI-1640 (Gibco) supplemented with 10% FBS. NK-92 cells were cultured in α-Minimum Essential Modified medium containing heat-inactivated 10% horse serum, 10% heat-inactivated FBS, and 200 U/ml recombinant human IL-2 (Beijing Four Rings Bio-Pharmaceutical). To successfully infect murine MC38 colon cancer cells with EV71, we constructed h-SCARB2-MC38 cells to overexpress the h-SCARB2 receptor that mediates EV71 infection. All cell lines were grown at 37°C in a humidified atmosphere containing 5% CO_2_ at 37°C with media containing 100 U/mL of penicillin and 100μg/mL of streptomycin.

The pcDNA3.1-2A, pcDNA3.1, p-EGFP-3C, and p-EGFP plasmid were provided by the Institute of Pathogen Microbiology, Chinese Academy of Medical Sciences. Recombinant human IFN-β (Catalog# 300-02BC) and recombinant human IFN-λ1 (Catalog# 300-02L) were purchased from PeproTech (Cranbury, NJ, United States).

The following antibodies were used in this study. Rabbit anti-PD-L1 polyclonal antibody (Catalog# PA5-28115) and rabbit anti-MICA/B polyclonal antibody (Catalog# PA5-109323) were purchased from Invitrogen. Rabbit-anti-human IL-28RA antibody (Catalog# HPA017319) was purchased from Atlas Antibodies. Mouse anti-Enterovirus 71 antibody (clone, 10F0) was purchased from Abcam and rabbit-anti-Enterovirus 71 VP1 antibody (Catalog# GTX132338) was purchased from GeneTex. Alexa Fluor 488-goat anti-rabbit IgG (H + L) antibody (Catalog # A-11008), Alexa Fluor 555-goat anti-rabbit IgG (H + L) antibody (Catalog# A-21429), and Alexa Fluor 488-goat anti-mouse IgG (H + L) antibody (Catalog# A-11001) as fluorescent-conjugated secondary antibodies were provided by Invitrogen. FITC-anti-human CD314 (NKG2D) antibody (Clone, 1D11), APC/Cyanine7-anti-human CD279 (PD-1) antibody (Clone, NAT105), PE-anti-human MICA/MICB antibody (Clone, 6D4), PE-anti-human CD274 (B7-H1, PD-L1) antibody (Clone, 29E.2A3), PE-anti-human IL-28RA antibody (Clone, MHLICR2a), PE-anti-mouse NK-1.1 antibody (Clone, S17016D), and PE/Dazzle™ 594-anti-mouse CD3 antibody (Clone, 17A2 CD3) were purchased from Biolegend. Fixable Viability Dye eFluor 506 (FVD 506) (Catalog# 65-0863-18) was purchased from eBioscience. Alexa Fluor^®^ 488 anti-human IL-10Rβ antibody (Clone, 90220) was purchased from R&D systems.

### Cell Infection, Transfection, and Stimulation

HT29 cells or Jurkat T cells were infected with EV71 at a multiplicity of infection (MOI) indicated in the figure legends and allowed to absorb for 2 h at 37°C. The inoculum was removed, and the cells were washed twice with medium to remove the unbounded viruses. Then the culture medium was added. The culture supernatants and infected cells were collected at different indicated times for testing. HT29 cells were transfected with pcDNA3.1-2A (control plasmid: pcDNA3.1) or p-EGFP-3C (control plasmid: p-EGFP) 2 μg using Lipofectamine 8000 (Beyotime Biotechnology, Catalog# C0533). The human IFN-β (10 ng/ml) or IFN-λ1(10 ng/ml) was used for IFNs treatment at the indicated time points. The poly(I:C) (4 μg/ml) was used to induce the production of IFNs at the time points indicated in the figure legends.

### Preparation of Intestinal Intraepithelial Lymphocytes

As previously described, the intestinal intraepithelial lymphocytes were isolated from the small intestine (Montufar-Solis and Klein, 2006). Firstly, the fatty connective tissue and Peyer’s patches of the small intestine were removed, then the small intestine was cut longitudinally, washed several times in 1 × PBS until clean, and then divided into small pieces of 2∼3 cm. The specimens were placed in pre-warmed iIEL digestive juice and incubated with stirring at 37°C for 40 min, followed by vigorous shaking for 20∼30 s. Then 1 × PBS was added to terminate the digestion, and the supernatants were passed through two nylon wool columns to remove undigested tissue fragments. The filtrate was collected and centrifuged at 400 g for 5 min, and the isolated cells were enriched by a discontinuous 40%/70% Percoll density gradient (GE Healthcare, Uppsala, Sweden) at 600 g for 25 min. Then the lymphocytes were harvested from the 40 to 70% Percoll interface. The viability, purity, and phenotype of iIELs were identified using FACS analysis.

### MACS Sorting of Cells

The intestinal intraepithelial lymphocytes from the C57BL/6 mouse were prepared, and then these cells were used to enrich NK1.1^+^iNK and CD3^+^iIELs by using the MojoSort™ Mouse NK Cell Isolation Kit (Biolegend, Catalog# 480049) and MojoSort™ Mouse CD3 Cell Isolation Kit (Biolegend, Catalog# 480024) respectively, according to the protocol provided by the manufacturer.

### Plaque Assays

Vero cells were grown into a monolayer at 24-well plates (1 × 10^5^ cells) and then infected with the 10-fold serial dilution of viral supernatant sample (100 μL/well) under 5% CO_2_ and 37°C. Then, we gently shook the well plate every 15 min to distribute the virus evenly. After 2 h, the Vero cells were washed by 1 × PBS and covered with 1mL of semisolid medium consisting of a 1:1 mixture of 2 × DMEM with 4% FBS and 2% methylcellulose solution. Depending on the virus analyzed, plaque formation may take 3 to 4 days. The semisolid medium was discarded by tipping, and cells were fixed and stained overnight with crystal violet staining solution (0.01% w/v) at room temperature. Finally, we observed and calculated the formation of plaques.

### Flow Cytometry

The cells were washed with 1 × PBS twice and stained with the primary antibody at 4°C for 40 min. The lymphocytes, harvested from the small intestine of mice, were blocked with an anti-mouse CD16/CD32 antibody (clone, 2.4G2) at room temperature for 10 min and then stained with antibodies at 4°C for 40 min. Finally, cells were washed with 1 × PBS twice and analyzed using a FACSCalibur flow cytometer (Gallios, Beckman Coulter, Inc., United Kingdom). FVD 506 was used to distinguish between living and dead cells. The median fluorescence intensity (MFI) was calculated in the subsequent analysis by FlowJo software (Treestar, Inc., Ashland, OR, United States).

### ELISA

The secretion levels of IFN-β, IFN-λ1, and IFN-λ2 proteins in the supernatant were detected by commercially available enzyme-linked immunosorbent assay kits, including Human IFN-β ELISA Kit (LIANKE, EK1236), Human IL-28A ELISA Kit (BOSTER, EK0969), and Human IL-29 ELISA Kit (BOSTER, EK0964).

### Immunofluorescence

The cells were fixed with 4% paraformaldehyde for 30 min at room temperature. After washing three times with 1 × PBS, the cells were permeabilized and blocked using 1% TritonX-100 in 5% BSA for 1 h. Then the cells were incubated with primary antibodies diluted in 2% BSA overnight at 4°C. The cells were washed three times with 1 × PBS and incubated with the appropriate fluorescence-conjugated secondary antibodies for 1 h at room temperature, followed by washed three times with 1 × PBS. The nuclei were counterstained with DAPI. Images were captured by Olympus confocal laser scanning microscope.

### Quantitative RT-PCR

The gene expression levels were determined by reverse quantitative real-time (qRT)-PCR. Total RNA was extracted by the Total RNA rapid extraction kit (Feijie, 220011) according to the protocol provided by the manufacturer. According to the manufacturer’s instructions, total RNA was reversely transcribed by HiFiScript cDNA Synthesis Kit to generate cDNA. The transcriptional levels of target genes were measured by qRT-PCR using the SYBR Green Real-time PCR Master Mix. GAPDH was used as an internal control. Primer sequences used are shown in Supplementary Table 1. The qRT-PCR amplification program was initially denaturation at 95°C for 10 min followed by 40 cycles of denaturation for 15 s at 95°C and then annealing for 30 s at 60°C.

### Cytotoxicity Assay

NK-92 cells were added to HT29 cells infected with EV71 at an MOI of 1 for 48 h, at effector/target (E:T) ratios of 5:1, 2.5:1, or 1.25:1. After incubated for 6 h, the medium was carefully obtained, and the LDH activity in the medium was determined using an LDH cytotoxicity assay kit (Beyotime Biotechnology; Catalog# C0017) following the manufacturer’s instructions. For blocking experiments, HT29 infected with or without EV71 were pre-incubated with saturating concentrations of anti-human MICA/MICB antibody (10 μg/ml; Biolegend; Clone, 6D4), anti-human PD-L1 antibody (10 μg/ml; Biolegend; Clone, 29E.2A3), or isotype-matched control antibody at 37°C for 2 h and then washed for use as effector cells. Then NK-92 cells were added, and the cytotoxicity was detected at an E:T ratio of 5:1 on 6 h by LDH cytotoxicity assay kit. The h-SCARB2-MC38 cells infected with EV71 at an MOI of 1 for 48 h were the target cells for assay of iNK and CD3^+^iIELs cell cytotoxicity. LDH was applied to analyze the susceptibility of EV71^–^h-SCARB2-MC38 and EV71^+^h-SCARB2-MC38 to iNK and CD3^+^iIELs lysis on 10 h at the E:T ratios of 40:1, 20:1, and 10:1. The absorbance of each well was measured at 490 nm/630 nm with a microplate auto reader (Bio-Rad). Cytotoxicity was calculated as follows: Percent lysis = (test sample - natural release)/(maximum release - natural release) *100%.

### Statistical Analysis

Results were expressed as mean**±** standard (SD). The statistical analysis was performed using Student’s *t*-tests, one-way analysis, or two-way analysis of variance (ANOVA) where appropriate. The *P*-value was calculated with the help of the software GraphPad Prism 8.0. Significant differences are indicated as *P < 0.05, ^**^P < 0.01, ^***^P < 0.001, and ^*⁣*⁣**^P < 0.0001.

## Results

### IFN-λ Inhibits EV71 Infection in Intestinal Epithelial Cells More Effectively Than IFN-β

**FIGURE 1:**
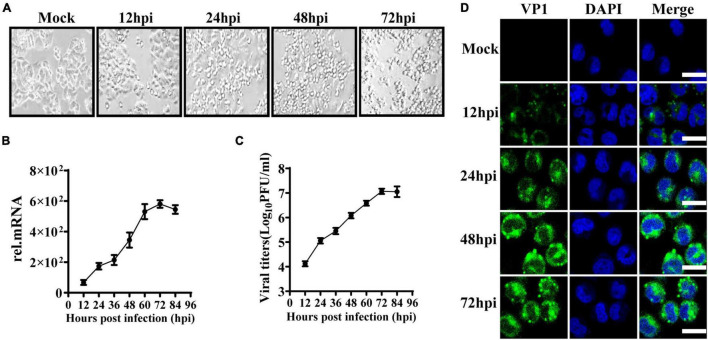
EV71 can replicate in human intestinal epithelial cells. **(A)** HT29 cells were inoculated with EV71 at an MOI of 1, and the images were obtained at the indicated time points. Representative images were shown. **(B)** The kinetic curve of EV71 replication in HT29 cells. HT29 cells and culture supernatants were harvested at indicated time points (MOI = 1). Total RNAs were extracted for reverse transcription (cDNA) to determine the mRNA level of EV71-VP1 using VP1 specific primers by qRT-PCR, and dates were calculated relative to GAPDH gene expression. **(C)** The cell culture supernatants were collected for virus titer detection by plaque assay. **(D)** HT29 cells were fixed in 4% paraformaldehyde at the indicated infection time points, then incubated with anti-EV71 VP1 antibody, followed by staining with Alexa Fluor 488-conjugated secondary antibody and DAPI to visualize the EV71 and nuclei. Olympus confocal laser scanning microscope was used for imaging. Mock was cells without infection. The results were presented from three independent experiments as mean ± SD.

To observe the innate immune response characteristics of IECs after EV71 infection, we used HT29 cells derived from the intestinal epithelium to study the replication dynamics and innate immune response characteristics. HT29 cells were infected with EV71 at a multiplicity of infection (MOI) of 1, and the cytopathic effect (CPE) was subsequently observed at indicated time points. At 24 h post-infection (hpi), the CPE appeared in EV71 infected cells and gradually aggravated (Figure 1A). Next, the virus growth curve was detected by quantitative real-time (qRT)-PCR and plaque assay. We found that the levels of EV71 replication in cells and culture supernatants were significantly increased in a time-dependent manner following EV71 infection (Figures 1B,C). To further visualize the replication and proliferation of EV71 in HT29 cells, EV71 was stained with an anti-VP1 antibody. As early as 12 hpi, the VP1 proteins were detected and increased significantly at 48 and 72 hpi (Figure 1D). Collectively, HT29 cells were susceptible to EV71 infection, and EV71 can effectively replicate in HT29 cells.

**FIGURE 2:**
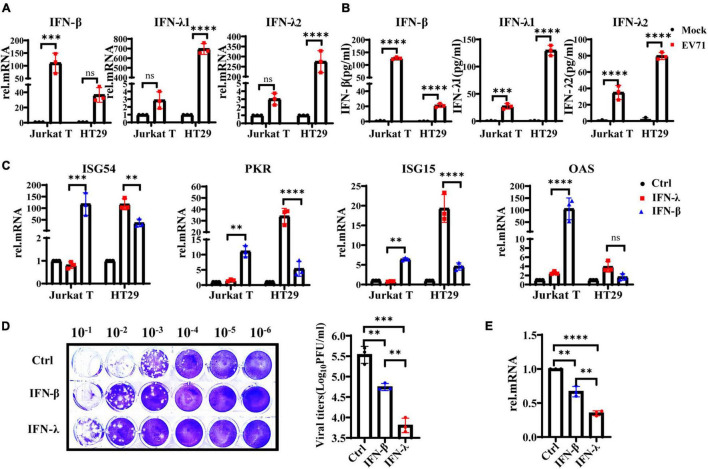
IFN-λ effectively inhibits EV71 infection in intestinal epithelial cells. **(A)** HT29 and Jurkat T cells were uninfected or infected with EV71 at an MOI of 1, qRT-PCR was used to detect transcriptional upregulation of both IFN-β, IFN-λ1, and IFN-λ2 at 48 hpi. **(B)** At the same infection conditions, the production and secretion of IFN-β, IFN-λ1, and IFN-λ2 proteins in the supernatant were detected by ELISA. Mock was cells without infection. **(C)** HT29 and Jurkat T cells were stimulated without or with 10 ng/ml recombinant protein IFN-β or IFN-λ1 for 12 h. Then the expression levels of ISG54, ISG15, PKR, and OAS were measured by qRT-PCR. Calculated the expression level of each gene relative to the expression of GAPDH and normalized it to mock-treated cells. Control was cells without treatment. **(D,E)** HT29 cells were pretreated with 10 ng/ml IFN-λ or 10 ng/ml IFN-β for 6 h, respectively, and then infected with EV71 at MOI of 1 for 24 h. The virus replication was monitored by qRT-PCR and plaque experiments. Control was cells with infected but without IFN treatment. Dates were presented as mean ± SD of three replicates (*n* = 3 independent experiments, ^**^P < 0.01, ^***^P < 0.001, ^*⁣*⁣**^P < 0.0001 and ns, not significant).

Interferons are essential in early antiviral infection. IFN-α/β family members are primary antiviral molecules, and almost any type of cell can secrete IFN-α/β after infection. IFN-λ was discovered in 2003. Initially, researchers believed that IFN-λ and IFN-α/β have similar functions via the induction of ISGs. However, many subsequent studies have found that IFN-λ has a unique role in antiviral and other immune responses (Jafarzadeh et al., 2021). Then we examined the production levels of IFN-λ1, IFN-λ2, and IFN-β in EV71-infected HT29 and Jurkat T cells by qRT-PCR and ELISA. The results showed that IFN-λ1 and IFN-λ2 were significantly induced in EV71-infected HT29 cells, and the production level was higher than IFN-β. By contrast, the production levels of IFN-λ1 and IFN-λ2 were extremely low, while the level of IFN-β was significantly higher in EV71-infected Jurkat T cells (Figures 2A,B). These results indicated that EV71 infection specifically stimulates the production of IFN-λs in IECs. To comparatively analyze the response of HT29 and Jurkat T to IFN-β or IFN-λ, respectively, we pretreated the cells with or without 10 ng/ml recombinant IFN-λ and IFN-β for 12 h and then detected the gene expression levels of ISG54, ISG15, PKR, and OAS by qRT-PCR. The results showed that both IFN-λ and IFN-β could stimulate the production of antiviral effectors ISG54, ISG15, and PKR. It is worth noting that IFN-λ treatment induces significantly higher ISG54, ISG15, and PKR than IFN-β in HT29 cells. By contrast, in Jurkat T cells, IFN-β stimulated higher levels of these genes, while IFN-λ had little effect. The results demonstrated that both lymphocytes and IECs were sensitive to IFN-β, while IFN-λ response was mainly limited to IECs (Figure 2C). Next, we investigated whether EV71 was sensitive to the antiviral effects of IFN-β and IFN-λ in an IFN-specific manner. HT29 was pretreated with recombinant IFN-β or IFN-λ for 6 h and then infected with EV71. The results showed that both IFN-β and IFN-λ could effectively inhibit virus infection, but IFN-λ displayed a stronger inhibitory effect on EV71 infection (Figures 2D,E). Altogether, the results strongly demonstrated that the IFN-λ was the key to effectively inhibiting EV71 replication in intestinal epithelial cells.

### EV71 Antagonizes the Antiviral Function of IFN-λ

**FIGURE 3:**
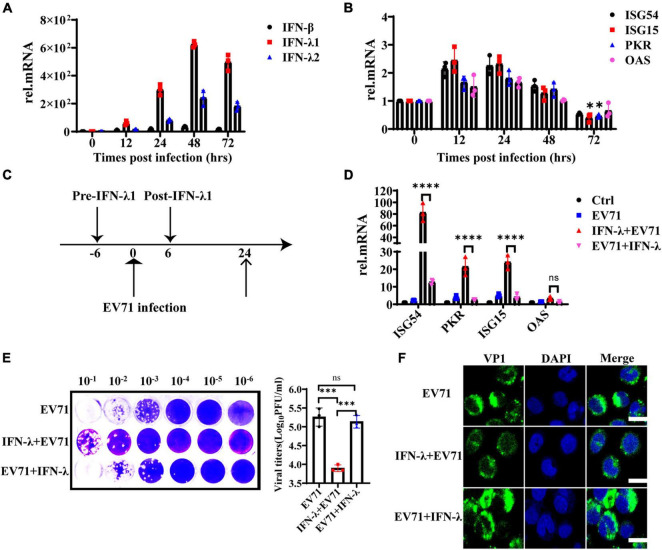
The antiviral function of IFN-λ is antagonized by EV71. **(A)** HT29 cells were infected with EV71 at an MOI of 1. The expression levels of IFN-β, IFN-λ1, and IFN-λ2 were detected at 0, 12, 24, 48, and 72 hpi by qRT-PCR. **(B)** At the same infection conditions, ISG54, ISG15, PKR, and OAS expression levels were detected by qRT-PCR. **(C)** Recombinant protein IFN-λ treatment (10 ng/ml) was performed 6 h before infection or 6 h after infection (MOI = 1). **(D)** After 24 hpi, the cellular RNA was extracted, and ISG54, ISG15, PKR, and OAS expression levels were measured by qRT-PCR. HT29 cells were mock-infected and were not treated by IFN-λ as “control (Ctrl)”. HT29 cells were infected with EV71 as “EV71”. The “IFN-λ + EV71” meant that HT29 was treated with IFN-λ 6 h before infection, and “EV71 + IFN-λ” meant that HT29 was treated with IFN-λ 6 h after infection. **(E)** The viral titers in the culture supernatant were monitored by plaque experiments at 24 hpi **(F)**. The intracellular viral protein (VP1) load was measured by confocal fluorescence microscopy. Dates were presented as mean ± SD (*n* = 3 independent experiments, *P < 0.05, ^***^P < 0.001, ^*⁣*⁣**^P < 0.0001 and ns, not significant).

HT29 cells were infected with EV71 at an MOI of 1, and the mRNA levels of IFN-β, IFN-λ1, and IFN-λ2 were detected at 0, 12, 24, 48, and 72 hpi to assess the continuous effect of EV71 infection on IFN-λ induction. As shown in Figure 3A, the gene expression levels of IFN-λ1 and IFN-λ2 continuously increased following viral infection and reached a peak at 48 h. As we know, the IFNs prevent viral infection by activating the transcription of a large number of antiviral effectors. To analyze the IFNs response during EV71 infection, we examined the production of downstream ISG54, ISG15, PKR, and OAS. The results showed that the mRNA levels of ISGs were up-regulated approximately 1∼2 folds at 12 and 24 hpi and began to decrease at 48 hpi, especially ISG15 and PKR even decreased to the basic level at 72 hpi compared with the uninfected cells (Figure 3B). These results indicated that EV71 infection induces the production of high levels of IFNs in HT29 cells, but the produced IFNs could not efficiently induce the production of ISGs. It was speculated that viruses or viral components accumulate in cells, blocking or weakening the response to interferon in the late stage of EV71 infection.

To investigate whether EV71 can antagonize the antiviral effect of exogenous IFN-λ, HT29 cells were infected with EV71 at an MOI of 1 and treated with 10 ng/ml recombinant protein IFN-λ at 6 h before infection or 6 h after infection separately, as indicated in Figure 3C. We found that IFN-λ pretreatment significantly induced the production of ISG54, ISG15, and PKR, while IFN-λ treatment after infection could not effectively activate their expression (Figure 3D). The virus titer was significantly inhibited when cells were pretreated with IFN-λ. However, the virus titer did not decrease significantly when the cells were treated with IFN-λ after infection (Figure 3E). These results were further confirmed by immunofluorescence assay of intracellular viral protein load (Figure 3F). It is indicated that EV71 can antagonize the antiviral effect of exogenous IFN-λ.

### 2Apro and 3Cpro of EV71 Antagonize the Production Levels of IFN-λ

**FIGURE 4:**
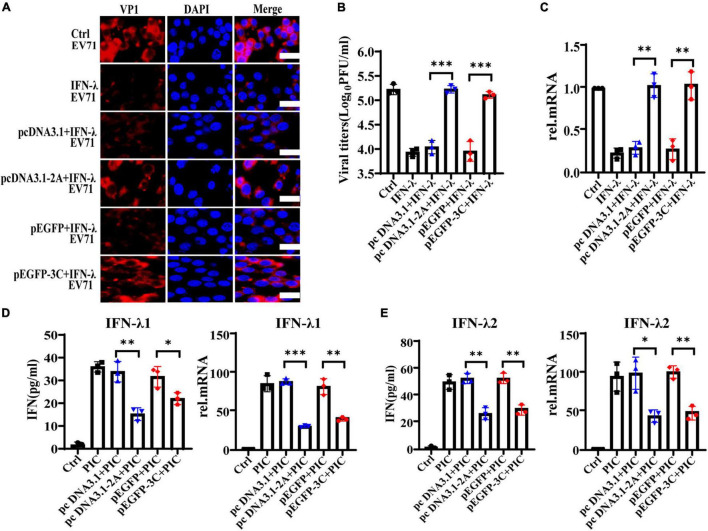
The 2Apro and 3Cpro of EV71 antagonize the antiviral function of IFN-λ and inhibit the expression level of IFN-λ induced by poly(I:C). HT29 cells were transfected with pcDNA3.1-2A (control plasmid: pcDNA3.1) or p-EGFP-3C (control plasmid: p-EGFP). After transfection 24 h, cells were treated with IFN-λ (10 ng/ml) 6 h before infection. Then cells were infected with EV71 at an MOI of 1. Cells and culture mediums were collected at 24 hpi. **(A)** The protein expression level of VP1 was determined by incubating with anti-EV71 VP1 antibody, followed by staining with Alexa Fluor 555-conjugated secondary antibody. **(B)** The mRNA expression levels of VP1 were determined by qRT-PCR. **(C)** Cell culture medium was prepared for virus titer analysis. Control was cells with infected only. IFN-λ group was cells infected and using IFN-λ pretreatment. **(D,E)** HT29 cells were transfected with pcDNA3.1-2A (pcDNA3.1) or p-EGFP-3C (p-EGFP), and after transfection 24 h, cells were treated with poly(I:C) (4 μg/ml). Cells and culture mediums were collected 24 h after stimulation. The protein expression levels of IFN-λ1 and IFN-λ2 were determined by ELISA, and the mRNA expression levels were determined by qRT-PCR. Control was cells without any treatment. PIC group was cells stimulated only by poly(I:C). Dates were presented as mean ± SD (*n* = 3 independent experiments, *P < 0.05, ^**^P < 0.01 and ^***^P < 0.001).

Previous studies demonstrate that the non-structural 2Apro and 3Cpro of EV71 are indispensable in maintaining the structural integrity of the virus and promoting the transcription and translation of viral proteins. They are extremely important functional proteins of enteroviruses. Importantly, it confirms that 2Apro and 3Cpro play a key role in the process of EV71 escaping from the host’s antiviral innate immunity. They can block and impair the transduction of multiple signal pathways such as RIG-I, MDA5, TLR3, NLRP3, and IFN-α/β, eventually inhibiting the production of IFN-α/β and ISGs. We transfected the plasmids pcDNA3.1-2A or p-EGFP-3C to overexpress 2Apro or 3Cpro and their control plasmids pcDNA3.1 or p-EGFP into HT29 cells, respectively. Then HT29 cells were treated with IFN-λ (10 ng/ml) for 6 h and infected with EV71 (MOI = 1) for 24 h, then cells and culture mediums were collected. We tested the cellular viral load and the supernatant viral titer and found that IFN-λ could significantly reduce the viral load and inhibit infection compared with the control. However, after overexpression of 2Apro or 3Cpro, the viral load and titer both increased markedly compared with the control plasmid (Figures 4A-C). In summary, the above results indicated that the 2Apro and 3Cpro of EV71 could antagonize the antiviral function of IFN-λ.

The intermediate dsRNA produced in the EV71 replication cycle can be recognized by PRRs such as MDA5 and TLR3 and induce the production of IFNs. As a synthetic dsRNA, poly(I:C) can mimic the dsRNA of EV71 and induce the production of IFNs. Therefore, PIC was used to simulate the dsRNA of EV71 in the follow-up to observe the effect of 2Apro and 3Cpro on interferons production. HT29 cells were transfected with pcDNA3.1-2A (pcDNA3.1) or p-EGFP-3C (p-EGFP), and at 24 h after transfection, cells were treated with PIC (4 μg/ml) for 24 h. We observed that 2Apro or 3Cpro significantly inhibited the production of IFN-λ1 and IFN-λ2 at both the gene and protein levels compared with the control plasmid, respectively (Figures 4D,E).

### The Expression Level of IFN-λR on Intestinal Epithelial Cells Is Inhibited by 2Apro and 3Cpro

**FIGURE 5:**
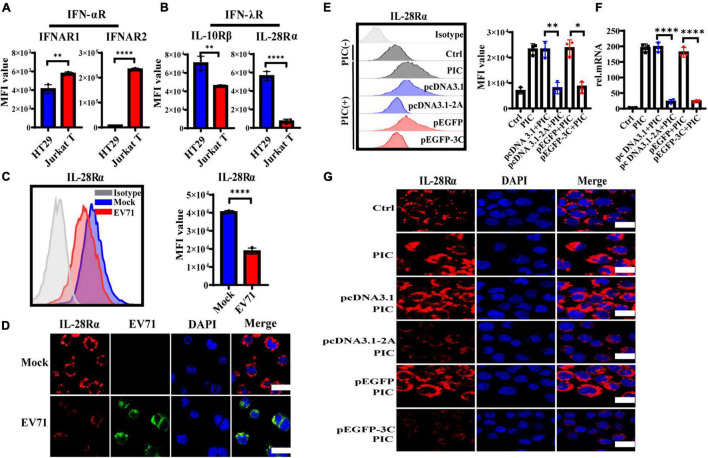
The expression level of IFN-λR on intestinal epithelial cells is inhibited by EV71, and its 2Apro and 3Cpro play an important inhibitory effect. **(A)** The expression levels of IFNAR1, IFNAR2, **(B)** IL-28Rα, and IL-10Rβ on HT29 or Jurkat T were measured by FACS. **(C)** HT29 cells were infected with EV71 at an MOI of 1. The membrane expression level of IL-28Rα was detected at 48 hpi by FACS. MFI, mean fluorescence intensity. **(D)** At 48 hpi, cells were fixed and stained with anti-EV71 (green) antibodies, anti-IL-28Rα (red) antibodies, and DAPI (blue) and examined by a confocal microscope. Mock was cells without infection. Merged images of the different channels were shown. **(E–G)** HT29 cells were transfected with pcDNA3.1-2A (pcDNA3.1) or p-EGFP-3C (p-EGFP). After 24 h transfection, the cells were treated with PIC (4 μg/ml) for another 24 h. Then cells were collected to detect the expression level of IL-28Rα by FACS and IF, and the mRNA expression level was determined by qRT-PCR. Control was cells without any treatment. PIC group was cells stimulated only by poly(I:C). Dates were presented as mean ± SD (*n* = 3 independent experiments, *P < 0.05, ^**^P < 0.01 and ^*⁣*⁣**^P < 0.0001).

IFNs initiate the antiviral response by binding to the corresponding specific receptors on the cell membrane. It has been shown that EV71 inhibited the cellular IFN-α/β response by reducing IFNAR1 levels (Lu et al., 2012). To address why EV71 caused IECs to poorly respond to the exogenous recombinant IFN-λ and antagonize the antiviral effect of IFN-λ, we explored the influence of EV71 on the expression of IFN-λR. We measured the expression level of IFNs receptors on HT29 and Jurkat T by flow cytometry (FACS). As expected, IFN-αR heterodimeric complex, including IFNAR1, and IFNAR2 was highly expressed on Jurkat T but lowered on HT29, which responded to IFN-α/β (Figure 5A). By contrast with Jurkat T, IFN-λR heterodimeric complex, including IL-28Rα, and IL-10Rβ was highly expressed on HT29 that was well known to respond to IFN-λ (Figure 5B), which explained why IECs responded strongly to IFN-λ, which was very important in the antiviral immune defense of IECs (Figure 2). Next, we examined the effect of viral infection on IL-28Rα at an MOI of 1. We found that the expression level of IL-28Rα on the membrane surface was significantly reduced at 48 hpi detected by FACS (Figure 5C). This phenomenon was also confirmed by confocal fluorescence microscopy (Figure 5D). The above results suggested that EV71 can inhibit the membrane expression of IFN-λR, thereby weakening the antiviral effect of IFN-λ.

HT29 cells were transfected with pcDNA3.1-2A (pcDNA3.1) or p-EGFP-3C (p-EGFP) to observe the effect of 2Apro and 3Cpro on the expression of IL-28Rα. After transfection 24 h, the cells were treated with PIC for another 24 h. It was found that PIC significantly up-regulated the expression of IL-28Rα at gene and protein levels. However, we found that both 2Apro and 3Cpro could markedly inhibit the expression level of IL-28Rα compared with the respective control plasmid (Figures 5E–G). These results indicated that the 2Apro and 3Cpro of EV71 can inhibit the expression level of IFN-λR on IECs to antagonize the antiviral ability of IFN-λ.

### The 2Apro and 3Cpro of EV71 Weaken the Response of IECs to IFN-λ

**FIGURE 6:**
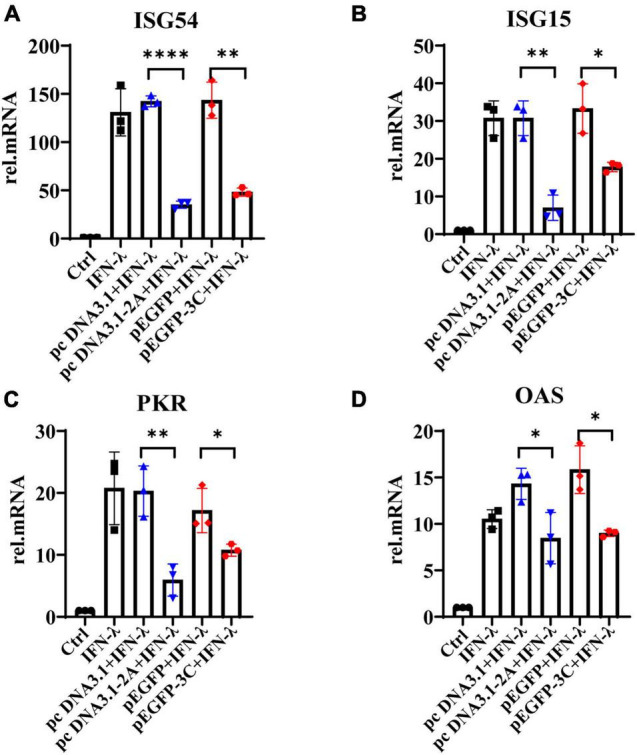
The 2Apro and 3Cpro of EV71 inhibit the production of antiviral proteins downstream of the IFN-λ pathway. HT29 cells were transfected with pcDNA3.1-2A (pcDNA3.1) or p-EGFP-3C (p-EGFP). After transfection 24 h, cells were stimulated by IFN-λ (10 ng/ml). The cells were collected 24 h later, and the mRNA expression levels of **(A)** ISG54, **(B)** ISG15, **(C)** PKR, and **(D)** OAS were measured by qRT-PCR. Control was cells without any treatment. Dates were presented as mean ± SD (*n* = 3 independent experiments, *P < 0.05, ^**^P < 0.01 and ^*⁣*⁣**^P < 0.0001).

We further observed that IFN-λ stimulated HT29 cells could induce the production of ISG54, ISG15, PKR, and OAS, but 2Apro and 3Cpro significantly inhibited their expression (Figure 6A–D). Based on the previous results, we believed that 2Apro and 3Cpro could reduce the expression level of IFN-λR on IECs, resulting in the inability of the IECs to recognize and respond to IFN-λ effectively, and ultimately hindered the downstream signal transduction of IFN-λ.

### EV71^+^ IECs Express Lower Levels of MICA/B and Higher Levels of PD-L1 and Are Less Susceptible to Innate Immune Cells Lysis

**FIGURE 7:**
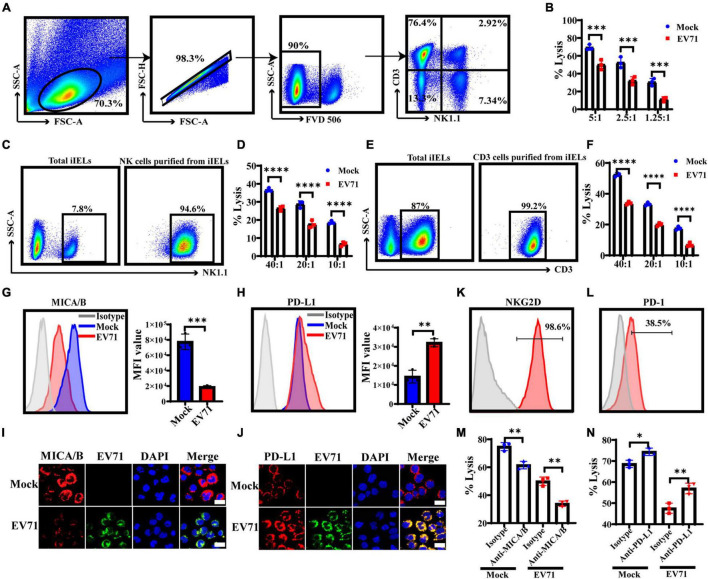
EV71^+^IECs express lower levels of MICA/B and higher levels of PD-L1 and are less susceptible to iNK and CD3^+^iIELs lysis. **(A)** The intestinal intraepithelial lymphocytes from the C57BL/6 mouse were separated and extracted, and the ratio of CD3^+^iIELs and NK1.1^+^iNK cells was analyzed by flow cytometry. **(B)** HT29 cells were infected with EV71 at an MOI of 1 for 48 h. LDH was applied to analyze the susceptibility of EV71^–^HT29 and EV71^+^HT29 to NK-92 cell lysis at 6 h at the indicated E:T ratios. **(C,E)** The intestinal intraepithelial lymphocytes were prepared to enrich iNK or CD3^+^iIELs using the MojoSort™ Mouse NK Cell Isolation Kit or the MojoSort™ Mouse CD3 T Cell Isolation Kit by MACS. Cells were stained with anti-NK1.1 antibody or anti-CD3 antibody. **(D,F)** The h-SCARB2-MC38 cells were infected with EV71 at an MOI of 1 for 48 h. LDH was applied to analyze the susceptibility of EV71^–^h-SCARB2-MC38 and EV71^+^h-SCARB2-MC38 to iNK or CD3^+^iIELs cell lysis on 10 h at the indicated E:T ratios. **(G,H)** HT29 cells were infected with EV71 at an MOI of 1. The membrane expression levels of MICA/B or PD-L1 were detected at 48 hpi by FACS. **(I,J)** At 48 hpi, cells were fixed and stained with anti-EV71 (green) antibodies, anti-MICA/B or PD-L1 (red) antibodies, and DAPI (blue) and examined by a confocal microscope. Mock was cells without infection. Merged images of the different channels were shown. **(K,L)** The expression levels of NKG2D or PD-1 on NK-92 were detected by FACS. **(M,N)** The EV71^–^HT29 and EV71^+^HT29 were pre-incubated with saturating concentrations of anti-MICA/B Ab (10 μg/ml) or anti-PD-L1 Ab (10 μg/ml), or isotype-matched control antibody at 37°C for 2 h and then washed for use as target cells. The NK-92 cells and treated HT29 were co-incubated for 6 h, and the cytotoxicity of NK-92 cells was detected at an E:T ratio of 5:1 on 6 h by LDH assay. Dates were presented as mean ± SD (*n* = 3 independent experiments, *P < 0.05, ^**^P < 0.01, ^***^P < 0.001, and ^*⁣*⁣**^P < 0.0001).

NK cells are an important component of the host’s innate immune defense system against bacterial or viral infections. They widely exist in intraepithelial, lamina propria of the small intestine, Payer patch, and mesenteric lymph nodes. Although the number of NK cells in the intraepithelial layer is very few, they have important antiviral functions. The CD3^+^iIELs are the most abundant lymphocytes in the intraepithelial layer, as shown in Figure 7A. CD3^+^iIELs express NKRs and exhibit NK-like killing activity (Zhou et al., 2007; Li et al., 2012, 2017). Therefore, it is of great significance to further study the role of iNK and CD3^+^iIELs in anti-EV71 infection. We found that the cytotoxicity of NK-92 against EV71^+^HT29 is much lower than EV71^–^HT29 (Figure 7B). This result suggested that EV71 decreased the susceptibility of HT29 to NK-92 cells cytolysis. The h-SCARB2-MC38 cell lines were established to verify the above phenomenon.

Furthermore, iNK and CD3^+^iIELs from the intestinal epithelial layer of normal C57BL/6 mice were separated and purified, and both purity > 90% (Figures 7C,E). Then the killing activity of iNK and CD3^+^iIELs against h-SCARB2-MC38 was tested, and we found that iNK and CD3^+^iIELs exhibited weaker cytotoxicity against the EV71^+^h-SCARB2-MC38 cells (Figures 7D,F). These results demonstrated that EV71^+^IECs were less susceptible to iNK and CD3^+^iIELs lysis.

Because NKG2D or PD-1 and its ligands play a key regulatory role in resisting viral infection, it is an important research route to study whether EV71 affects the expression of NKG2DL or PD-L1 on IECs and helps to evade iNK and CD3^+^iIELs lysis. Our result showed that the expression level of MICA/B on the surface of EV71^+^HT29 cells was down-regulated, but the PD-L1 was significantly up-regulated (Figures 7G,H). Immunofluorescence results also confirmed the above phenomenon (Figures 7I,J). The FACS result showed that NK-92 cells both expressed NKG2D and PD-1 (Figures 7K,L). These results indicated that the expression levels of MICA/B and PD-L1 on IECs changed significantly when EV71 infection occurred, the activation signal was weakened, and the inhibitory signal was enhanced, resulting in NK cells being unable to exert their antiviral effects effectively. To confirm this, after blocking MICA/B on EV71^+^HT29 with an anti-MICA/B antibody, the cytolytic activity of NK cells decreased significantly compared with isotype control (Figure 7M). After blocking PD-L1 on EV71^+^HT29 with an anti-PD-L1 antibody, the cytolytic activity of NK cells increased significantly compared with isotype control (Figure 7N). These results suggest that the lytic activity of NK against IECs was correlated with the interaction between NKG2D or PD-1 and its ligands. EV71 infection could reduce the susceptibility of IECs to iNK and CD3^+^iIELs lysis by down-regulation of MICA/B and up-regulation of PD-L1.

### 2Apro and 3Cpro Suppress NKG2DL Expression and Up-Regulate PD-L1 Expression on IECs

**FIGURE 8:**
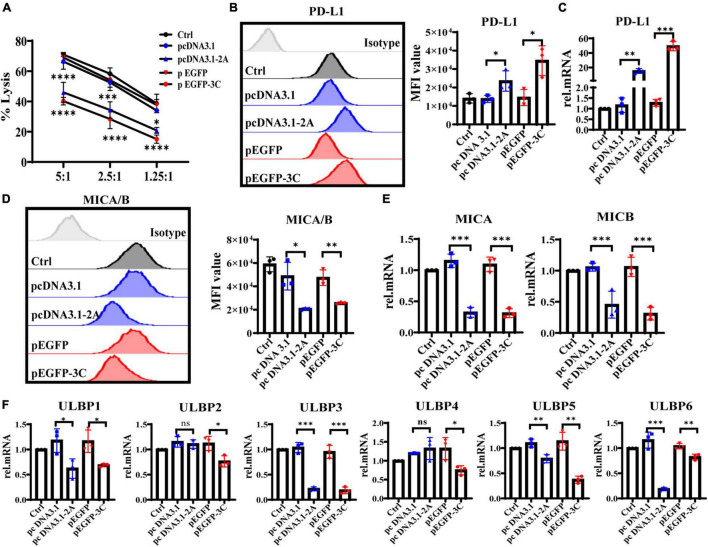
The 2Apro and 3Cpro reduce the susceptibility of IECs to NK lysis by suppressing NKG2DL expression and improving PD-L1 expression on IECs. **(A)** Analyze the susceptibility of HT29 cells transfected with the pcDNA3.1-2A (pcDNA3.1) or p-EGFP-3C (p-EGFP) plasmids to NK-92 cell lysis on 6 h at the indicated E:T ratios by LDH. **(B,D)** HT29 cells were transfected with the four kinds of plasmids. After 24 h transfection, the cells were collected to detect the expression levels of PD-L1 and MICA/B by FACS. **(C,E,F)** The mRNA expression levels of PD-L1, MICA/B, and ULBP1∼6 were determined by qRT-PCR. Control was cells without any treatment. Dates were presented as mean ± SD (*n* = 3 independent experiments, *P < 0.05, ^**^P < 0.01, ^***^P < 0.001, ^*⁣*⁣**^P < 0.0001 and ns, not significant).

To further explore whether 2Apro and 3Cpro of EV71 affected the expression levels of NKG2DL and PD-L1, eventually reducing the susceptibility of IECs to iNK and CD3^+^iIELs lysis, we analyzed the cytolytic activity of NK-92 to HT29 that was transfected with 2Apro and 3Cpro. At the different effective target ratios, 2Apro and 3Cpro remarkably decreased cytotoxicity of NK-92 against HT29 cells (Figure 8A). To further confirm that the expression level of MICA/B decreased significantly, while the expression level of PD-L1 increased significantly on HT29 cells after being transfected with 2Apro and 3Cpro (Figures 8B-E). Furthermore, we also tested the changes of ULBP1∼6 and found that 2Apro and 3Cpro also generally inhibited the expression level of the ULBP family (Figure 8F). In light of these data, we proved that 2Apro and 3Cpro inhibited the expression level of NKG2DL and promoted the expression level of PD-L1 to promote EV71 to evade the iNK and CD3^+^iIELs immunosurveillance in the intestinal mucosal barrier.

## Discussion

The intestine is not only the digestive organ but also the body’s largest immune organ. Intestinal mucosal immunity requires a timely immune response against invading pathogens to maintain intestinal homeostasis and protect the intestine from foreign pathogens. The single layer of intestinal epithelial cells and intraepithelial lymphocytes located between the epithelial cells form the first line of defense against foreign pathogen infection (Kansler and Li, 2019; Ma et al., 2021). Epithelial cells can respond quickly to antigen stimulation and remove foreign pathogens. At the same time, intraepithelial lymphocytes can kill infected epithelial cells and limit pathogen spreading. As an enterovirus, EV71 is transmitted by the intestinal tract; however, the mechanisms it uses to evade the immunosurveillance of the intestinal mucosal immune system are still incompletely clarified.

IECs are located in the outermost layer of the intestinal mucosa, performing the first line of defense against foreign pathogen infection. Studies have shown that IFN-λ can effectively limit enterovirus infection in intestinal epithelial cells, such as rotavirus, reovirus, and norovirus (Nice et al., 2014; Hernandez et al., 2015; Mahlakoiv et al., 2015). It has been reported that reovirus did not replicate in IECs after infecting IFN-α/β receptor-deficient mice (Ifnar1^–/–^) by gavage and, strikingly, did productively replicate in the lamina propria. However, it did replicate exclusively in IECs in IFN-λ receptor-deficient mice (Ifnlr1^–/–^), suggesting that the gut mucosa is armed with a compartmentalized IFN system in which epithelial cells mainly respond to IFN-λ for antiviral defense (Mahlakoiv et al., 2015). Our study revealed that IECs infected by EV71 mainly induced IFN-λ production, and IFN-λ inhibited EV71 infection more significantly than IFN-β (Figure 2). It further provided evidence for the important role of IFN-λ in anti-enterovirus infection. Members of the IFN-α/β family are key antiviral molecules. However, due to the ubiquitous expression of their receptors throughout the body, they can cause severe inflammatory factor storms and aggravate the development of the disease. By contrast, IFN-λR (a heterodimer composed of IL-28Rα and IL-10Rβ chains) highly restricted expresses on the mucosal surface of the gastrointestinal tract mainly, so the antiviral response of IFN-λ is limited to the mucosal surface of the intestine (Pott and Stockinger, 2017). Treatment with IFN-λ can eliminate the enterovirus without causing excessive inflammation. Therefore, the application of IFN-λ in the treatment of EV71 infection will be of great significance.

IFNs bind to their receptor to activate signal transduction and then stimulate ISGs to exert their antiviral function. It has been proven that 2Apro of EV71 interferes with type I IFN signaling by reducing the expression level of IFNAR1 in host cells and ultimately weakens response to IFN-α/β, which is an effective mechanism for EV71 to evade innate antiviral responses (Lu et al., 2012). We demonstrated that EV71 induced high levels of IFN-λ but only moderately stimulated the production of ISGs in the early stages of infection and significantly reduced ISGs expression to baseline levels in the later stages of infection. Treatment with exogenous IFN-λ before EV71 infection can effectively induce the production of ISGs and markedly reduce the damage of IECs. However, treatment with IFN-λ after EV71 infection could not effectively induce the production of downstream ISGs and could not inhibit infection (Figure 3).

Furthermore, we found that EV71 2Apro and 3Cpro inhibit the expression level of IFN-λR on IECs, thus contributing to antagonizing the antiviral ability of IFN-λ (Figure 5), which is the first finding demonstrating that 2Apro and 3Apro of EV71 antagonize antiviral innate immune response by suppressing IFN-λ production of IECs and attenuating the response of IECs to IFN-λ possibly by inhibiting the membrane expression of IFN-λR and reducing ISGs induction. Of course, EV71 infection could disrupt antiviral immunity at multiple molecular levels. EV71 has been reported to evolve different strategies to attenuate antiviral responses and facilitate replication inside the host cells. EV71-encoded proteases could antagonize RIG-I, MDA5, and TLRs signaling pathways to inhibit the production of type I IFNs. It can also inhibit the expression level of type I IFN receptor and JAK/STAT signaling, weakening the response to type I IFNs and inhibiting ISGs induction (Pathinayake et al., 2015; Rasti et al., 2019). Accordingly, EV71 2A and 3C proteases have a pleiotropic role in antiviral signaling. Intriguingly, in the local intestinal mucosal system, IFN-λ plays a more important role in host antiviral response. The evasion strategies of EV71 from IFN-λ-mediated antiviral responses in the local intestinal mucosal barrier contribute to the invasion, replication, and spreading of EV71 and the pathogenesis of EV71 infection.

The CD3^+^iIELs and iNK cells exist in the intestinal mucosal immune system, contain cytoplasmic granules, express a variety of NKRs, identify the infected IECs in time, and exert NK-like killing activity. Therefore, it is of great significance to further study the role of iNK and CD3^+^iIELs in anti-EV71 infection. Our research had proved that EV71 reduced the susceptibility of IECs to iNK and CD3^+^iIELs lysis. According to reports, many viruses can evade host antiviral immune responses by inhibiting the expression of activating ligands such as NKG2DL. For example, various proteins encoded by human cytomegalovirus (CMV) can degrade NKG2DL and prevent its expression on the cell surface. The UL16 protein encoded by CMV can inhibit the expression of MICB, ULBP1, ULBP2, and ULBP6. The US18 and US20 proteins can inhibit the expression of MICA (Fielding et al., 2014). The UL142 protein can markedly reduce the expression of MICA and ULBP3 and compromise the efficacy of NK cell cytotoxicity (Ashiru et al., 2009; Bennett et al., 2010). HIV-encoded Nef protein can inhibit the expression of MICA, ULBP1, and ULBP2 (Cerboni et al., 2007). Due to the key regulatory role of NKG2D or PD-1 and its ligands in the defense against virus infection, it is an important research route to determine whether EV71 affects the expression of NKG2DL or PD-L1 on IECs and contributes to evading iNK and CD3^+^iIELs lysis. Our study found that the expression level of NKG2DL on the surface of HT29 was significantly inhibited, while the expression level of PD-L1 was significantly increased after being infected by EV71 (Figure 7). It was further discovered that the 2Apro and 3Apro of EV71 can markedly inhibit the expression of NKG2DL and promote the expression of PD-L1, allowing intestinal epithelial cells to evade the killing of NK cells (Figure 8).

**FIGURE 9:**
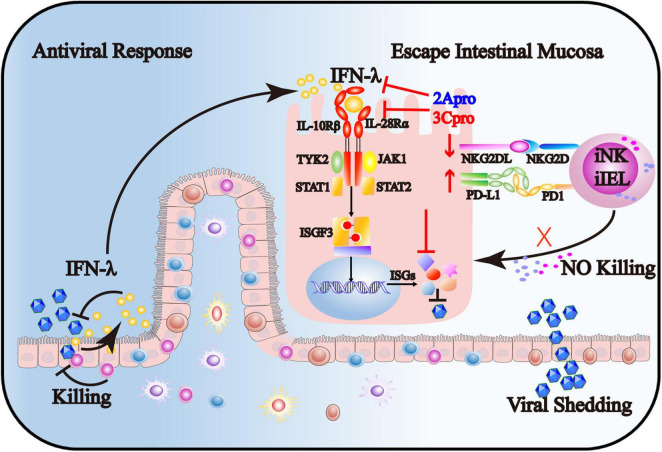
The 2Apro and 3Cpro of enterovirus 71 inhibit the IFN-λ production and IFN-λ receptor expression and further decrease the response of IECs to IFN-λ. Also, 2Apro and 3Cpro reduce the susceptibility of IECs to iNK and CD3^+^iIELs lysis, possibly by down-regulation of NKG2DL and up-regulation of PD-L1.

In summary, our findings provide novel mechanisms for EV71 to evade intestinal mucosal immune system surveillance, including IECs, iNK, and CD3^+^iIELs. As shown in Figure 9, on the one hand, 2Apro and 3Cpro of enterovirus 71 inhibit the IFN-λ production and IFN-λ receptor expression and further decrease the response of IECs to IFN-λ. On the other hand, 2Apro and 3Cpro reduce the susceptibility of IECs to iNK and CD3^+^iIELs lysis, possibly by down-regulation of NKG2DL and up-regulation of PD-L1. Targeted interfering with 2Apro and 3Cpro may be an important therapeutic strategy for EV71 intestinal infection.

## Data Availability Statement

The raw data supporting the conclusions of this article will be made available by the authors, without undue reservation.

## Author Contributions

YD conceived and designed the study, performed most of the experiments, and wrote the manuscript draft. JL contributed to the experimental materials and methodology, and directed and perform some experiments. NL provided suggestions, analyzed, and discussed the data. CZ contributed to the conception and design of the study, directed the research program, analyzed and discussed the data, and wrote and edited the manuscript. All authors read and approved the final version of the manuscript.

## Conflict of Interest

The authors declare that the research was conducted in the absence of any commercial or financial relationships that could be construed as a potential conflict of interest.

## Publisher’s Note

All claims expressed in this article are solely those of the authors and do not necessarily represent those of their affiliated organizations, or those of the publisher, the editors and the reviewers. Any product that may be evaluated in this article, or claim that may be made by its manufacturer, is not guaranteed or endorsed by the publisher.
