# Supplementary material for: Enterovirus 71 Antagonizes Antiviral Effects of Type III Interferon and Evades the Clearance of Intestinal Intraepithelial Lymphocytes
Source: Front Microbiol. 2022 Feb 1;12:806084. doi: 10.3389/fmicb.2021.806084 (PMC8848745; doi:10.3389/fmicb.2021.806084)
Supplement: Supplementary file 1 [file Table_1.DOCX]

Supplementary Material

| **Gene name** | **Primer sequence（5'-3'）** | **bp numbers** |
| --- | --- | --- |
| GAPDH | Forward-GGAGCGAGATCCCTCCAAAAT | 21 |
|  | Reverse-GGCTGTTGTCATACTTCTCATGG | 23 |
| ISG54 | Forward-GGAGCAGATTCTGAGGCTTTGC | 22 |
|  | Reverse-GGATGAGGCTTCCAGACTCCAA | 22 |
| ISG15 | Forward-AGCAGCTCCATGTCGGTGTCA | 21 |
|  | Reverse-CAGAGGTTCGTCGCATTTGTCC | 22 |
| PKR | Forward- GAAGTGGACCTCTACGCTTTGG | 22 |
|  | Reverse- TGATGCCATCCCGTAGGTCTGT | 22 |
| OAS | Forward-TAATAAGTGACCGTGACCCACA | 22 |
|  | Reverse-GCATCTTTGCATCTACCTCGA | 21 |
| MICA | Forward-GGGAATCACAGCACTCACC | 19 |
|  | Reverse-TTCTTCTTACAACAACGGACA | 21 |
| MICB | Forward-GGAATGGAACCTACCAGACCTG | 22 |
|  | Reverse-CTGTCCGTTGACTCTGAAGCAC | 22 |
| ULBP1 | Forward-TTCCTTCTGTGCCTCCCG | 18 |
|  | Reverse-GCCTTGGGTTGGGTTGTG | 18 |
| ULBP2 | Forward-ATCTTGGCAGTTCAGTTTCG | 20 |
|  | Reverse-ACCTTGTCATTCTCCCACTTT | 21 |
| ULBP3 | Forward-GCAGGTCAGGATGTCTTGTG | 20 |
|  | Reverse-GAAGGTGGTCAGTCCGCTA | 19 |
| ULBP4 | Forward-TCAAACCTCTGGGCCTCCTG | 20 |
|  | Reverse-CTCCATTGGTGGCGAACTGC | 20 |
| ULBP5 | Forward-ATCACCGTCATCCCTAAGTT | 20 |
|  | Reverse-ACTGGGTTCTGTGCTTTCC | 19 |
| ULBP6 | Forward- AGAGCAACTGCTTGACATTCAGC | 23 |
|  | Reverse- GAGTAGGAAGGTCTGTCCATCG | 22 |
| PD-L1 | Forward-TGCCGACTACAAGCGAATTACTG | 23 |
|  | Reverse-CTGCTTGTCCAGATGACTTCGG | 22 |
| IFN-β | Forward-GACCAACAAGTGTCTCCTCCAAA | 23 |
|  | Reverse-GAACTGCTGCAGCTGCTTAATC | 22 |
| IFN-λ1 | Forward- AACTGGGAAGGGCTGCCACATT | 22 |
|  | Reverse- GGAAGACAGGAGAGCTGCAACT | 22 |
| IFN-λ2 | Forward-TCGCTTCTGCTGAAGGACTGCA | 22 |
|  | Reverse-CCTCCAGAACCTTCAGCGTCAG | 22 |
| IL-28Rα | Forward-CAGCAAGTTCTCTAAGCCCACC | 22 |
|  | Reverse-GTCATTCACGGACTCTGGTCTG | 22 |
| EV71-VP1 | Forward-GCAGCCCAAAAGAACTTCAC | 20 |
|  | Reverse-ATTTCAGCAGCTTGGAGTGC | 20 |

**Table S1|Real-time PCR primers.**
